# Femoral ontogeny in humans and great apes and its implications for their last common ancestor

**DOI:** 10.1038/s41598-018-20410-4

**Published:** 2018-01-31

**Authors:** Naoki Morimoto, Masato Nakatsukasa, Marcia S. Ponce de León, Christoph P. E. Zollikofer

**Affiliations:** 1 Laboratory of Physical Anthropology, Graduate School of Science, Kyoto University, Kyoto, Japan; 2 Anthropological Institute and Museum, University of Zurich, Zurich, Switzerland

## Abstract

Inferring the morphology of the last common ancestor of humans, chimpanzees and gorillas is a matter of ongoing debate. Recent findings and reassessment of fossil hominins leads to the hypothesis that the last common ancestor was not extant African ape-like. However, an African great-ape-like ancestor with knuckle walking features still remains plausible and the most parsimonious scenario. Here we address this question via an evolutionary developmental approach, comparing taxon-specific patterns of shape change of the femoral diaphysis from birth to adulthood in great apes, humans, and macaques. While chimpanzees and gorillas exhibit similar locomotor behaviors, our data provide evidence for distinct ontogenetic trajectories, indicating independent evolutionary histories of femoral ontogeny. Our data further indicate that anthropoid primates share a basic pattern of femoral diaphyseal ontogeny that reflects shared developmental constraints. Humans escaped from these constraints via differential elongation of femur.

## Introduction

Bipedal locomotion with an upright posture is a defining feature of hominins (species more closely related to humans than to chimpanzees). This stands in contrast to the diversified, quadrupedal locomotor behaviors of extant great apes^1–4^. Using the extant great apes as a model, various locomotor modes have been proposed as ancestral states of human bipedal locomotion. One of these is the so-called “knuckle walking,” a peculiar mode of terrestrial quadrupedal locomotion performed by African great apes (*Pan* and *Gorilla* species and subspecies)^5^. The knuckle-walking hypothesis posits that this mode of locomotion was characteristic of the last common ancestor of gorillas, chimpanzees and humans (GCH-LCA), while vertical climbing, performed by all of the extant great apes, is regarded as the locomotor mode precedent to knuckle walking^6^. Alternatively, the orangutan has been proposed as a model for the evolution of bipedality. This hypothesis posits that human bipedality evolved from a generalized quadrupedal, and hand-assisted arboreal, repertoire of locomotion^7^. In contrast to both hypotheses, phyletic and functional analyses of the fossils of *Ardipithecus ramidus*^8–12^ provide evidence that hominin bipedality might have evolved from a mode of locomotion no longer present in extant great apes (“careful climbing, clambering, and bridging”^11^).

Attempts at reconstructing the mode of locomotion of the last common ancestor of gorillas, chimpanzees, and humans (GCH-LCA) are mainly based on comparative behavioral and skeletal data of living and fossil apes. The femur has been shown to be a key element for tracing evolutionary shifts in locomotor modes^13^. It provides attachment sites for various muscles that are of special relevance for taxon-specific locomotor behaviors such as *gluteus maximus*^14–16^. Accordingly, evolutionary changes in femoral morphology reflect changes in the musculoskeletal biomechanics of the hind limbs. A recent comparative study of the proximal femoral morphology of Miocene apes, *Orrorin tugenensis*, Plio-Pleistocene hominins, and extant hominoids (humans and apes) supports the view that phenotypic variation in extant hominoids is the result of taxon-specific specialization and evolutionary divergence from the primitive state of Miocene apes that is no longer present in extant forms^13^.

Amidst these controversies, a central question remains: do the femoral morphologies of chimpanzees and gorillas indicate a knuckle-walking LCA, or do they indicate convergent evolution of knuckle walking from a generalized LCA? Answering this question permits inferences on evolutionary scenarios of human bipedality. However, resolving the knuckle-walking controversy is challenging, since phenotypic variation not only reflects taxon-specific adaptations, but also phyletic history, and individual locomotor behaviors. Taxon-specific adaptations and phyletic history tend to constrain phenotypic variability via the genetically determined developmental program^17,18^, while actual behavioral patterns tend to produce patterns of phenotypic variability not coded in the developmental program but reflecting actual environmental influences. To discriminate between these effects, we use here an evolutionary developmental approach. Specifically, we compare taxon-specific ontogenetic trajectories of the femoral diaphysis in humans, great apes (chimpanzees, gorillas, and orangutans), and Japanese macaques from birth to adulthood, using methods of geometric morphometrics. The evolutionary developmental approach is of special relevance for two reasons. First, it provides insights into how new taxon-specific morphologies emerge via the evolutionary modification of ancestral developmental programs. Second, tracking developmental trajectories from birth to adulthood permits to assess the relative importance of genetic versus environmental factors during the formation of the phenotype^17,19^. For example, investigating neonatal individuals permits to determine the role of genetic effects by excluding the effects of *in vivo* locomotor behaviors during postnatal life^17,19–21^.

First, we ask how chimpanzee- and gorilla-specific features of the femur are brought about during development. Ontogenetic commonalities and differences between these taxa can be used to test the “knuckle-walking ancestor” hypothesis, that is, to assess whether femoral morphologies of chimpanzees and gorillas can be explained by shared knuckle-walking ancestry or not. Given the importance for hind limb-mediated body propulsion^16^, patterns of variability of the femoral morphology are expected to reflect adaptive evolution and/or *in vivo* modification. Thus, under the knuckle-walking hypothesis, chimpanzees and gorillas should have a conserved GCH-LCA pattern of femoral morphology and ontogeny due to constraints of stabilizing selection, and/or *in vivo* locomotor behavior. On the other hand, under the “generalized quadruped ancestor” hypothesis, morphological and developmental similarities among chimpanzees and gorillas should result from convergent evolution of knuckle-walking. Under this hypothesis, the modes of knuckle-walking could actually be different in terms of biomechanics as proposed by Kivell and Schmitt^22^. Based on these considerations, the following patterns of variation in femoral morphology and ontogeny are expected in humans, great apes and Japanese macaques for each hypothesis.

- Under the “knuckle-walking ancestor” hypothesis, the expectation is that chimpanzee and gorilla femoral morphologies should be more similar to each other than to orangutans (and humans) already at birth. Also, developmental trajectories should have similar directions. Any differences between chimpanzee and gorilla femoral morphologies should largely reflect allometric (*i.e*., body size-related) differences. Specifically, gorillas should exhibit an extended version of the chimpanzee developmental trajectory, reflecting their larger body size.
- Under the “generalized quadruped ancestor” hypothesis, the expectation is that femoral morphologies and developmental trajectories of chimpanzee and gorilla should be distinct from those of Japanese macaques, a generalized quadruped. The difference between gorilla and chimpanzee neonate femoral morphologies should also be similar to the difference between any of them and orangutans or humans. Second, chimpanzee and gorilla femoral morphologies are expected to be more different from each other at birth than at adulthood. Alternatively, if chimpanzees and gorillas engage in different modes of knuckle-walking, chimpanzees and gorillas could exhibit divergent ontogenetic trajectories indicating that their developmental patterns diverged from the pattern of GCH-LCA in taxon-specific ways.

Second, we ask how human-specific features of the femur are brought about during development. Lovejoy *et al*. suggested that the key features of the human postcranial skeleton associated with bipedality such as pelvic shape and the presence of a lateral patellar lip on the femur should be interpreted in a developmental framework^23^. Various studies have documented that some human-specific features of the femur are genetically determined while others are environmentally induced. For example, the lateral patellar lip^24,25^ is present already at birth^26^ and develops further during postnatal life^25^, whereas the bicondylar angle (*i.e*., the inclination of the femoral shaft relative to the knee joint) only develops through postnatal ontogeny as an *in vivo* response to bipedal locomotion^21,27,28^. In this study, we investigate a principal feature of the human femur; its slender shape. Compared to great apes, human hind limbs are elongated relative to body weight, and long bones exhibit a gracile shape (small cross-sectional circumference relative to length^29–31^). Thus, humans should exhibit a peculiar pattern of femoral diaphyseal development distinct from great apes, and great apes should be more similar to each other than to humans. Various experimental studies have shown that elongated hind limbs contribute to reduction of locomotor cost during bipedal walking^32–34^. The elongated hind limb is a defining feature of our genus *Homo*, but recent findings and reassessment of *Australopithecus* fossils show that hind limb elongation relative to body weight predates the emergence of *Homo*^35^. This suggests a deeper root of hind limb elongation than previously thought^35^. We ask how the developmental program of the LCA had been modified to realize elongated hind limb of humans, compared to extant great apes.

## Materials and Methods

### Sample

The sample consists of femora of humans (*Homo sapiens*, *N* = 132), chimpanzees (*Pan troglodytes, N* = 70), gorillas (*Gorilla gorilla, N* = 51), orangutans (*Pongo pygmaeus, N* = 39), and Japanese macaques (*Macaca fuscata*, *N* = 21), documenting ontogeny from late fetal to adult stages (pooled sex; see Supplementary Table S1 for the detailed sample structure). The sample includs neonatal specimens with documented age at death (*N* = 1 for each taxon; *Homo sapiens*: 40 gestational weeks, *Pan troglodytes*: 5 days post partum, *Gorilla gorilla*: 2 minutes post partum, *Pongo pygmaeus*: stillbirth, *Macaca fuscata*: 3 days post partum). These specimens were used as references defining the onset of postnatal development for each taxon. Most of the human and great ape specimens were obtained from the collection of the Anthropological Institute and Museum of the University of Zurich. The neonatal specimen of gorilla was obtained from the collections of the Smithsonian Institution National Museum of Natural History. The Japanese macaque specimens were obtained from the collections of Primate Research Institute of Kyoto University. None of the specimens used in this study exhibited macroscopic pathologies.

### Volumetric data acquisition

Femora of large specimens (femoral length ≥150 mm) were scanned using a Siemens 64-detector-array CT device (for humans, chimpanzees, gorillas, and orangutans) or a Toshiba 16-detector-array CT device (for Japanese macaques) with the following data acquisition and image reconstruction parameters: Siemens: beam collimation: 1.0 mm; pitch: 0.5–0.75; image reconstruction kernel: standard/sharp (B30s/B70s); slice increment: 0.3–0.5 mm; Toshiba: beam collimation: 1.0 mm; pitch: 0.75–0.875; image reconstruction kernel: standard/sharp (FC03/FC30); slice increment: 0.2–0.4 mm. This resulted in volumetric data sets with an isotropic spatial resolution in the range of 0.3–0.5 mm. Small specimens (femoral length <150 mm) were scanned using micro-CT scanners [µCT80, Scanco Medical (Switzerland) for humans, chimpanzees, gorillas, and orangutans (isotropic voxel resolution: 75 µm), and ScanXmateA080S, Comscantecno (Japan) for Japanese macaques (isotropic voxel resolution: 75–80 µm)]. All specimens of the femur were considered as right.

### Morphometric data acquisition and analysis

#### Morphometric data acquisition

We use a landmark-free approach, that is, morphometric mapping^36–39^, which is suitable for the analysis of the “featureless” morphologies of long bone diaphyses. In immature specimens, unfused epiphyses are often missing, or their position relative to the diaphysis cannot be reliably reconstructed in dry-skeleton specimens (Supplementary Fig. S1). We therefore focus here on diaphyseal morphology. The femoral diaphysis was extracted from the CT volumetric data using the epiphyseal lines as proximal and distal delimiters. The CT data were then used to determine the centroid line of the diaphysis, and serial diaphyseal cross sections were re-sampled along this line. To reduce noise, subperiosteal (external) outlines of each cross-section were parameterized with elliptical Fourier analysis (EFA)^40^. For each specimen, measurements of the external diaphyseal radius (*r*_*ext*_) were sampled around each cross-sectional outline, and along the entire diaphyseal shaft. The entire femoral diaphyseal shape was thus quantified by densely sampling the morphometric variable (*r*_*ext*_) rather than a set of predefined characters.

#### Morphometric analysis

**Figure 1:**
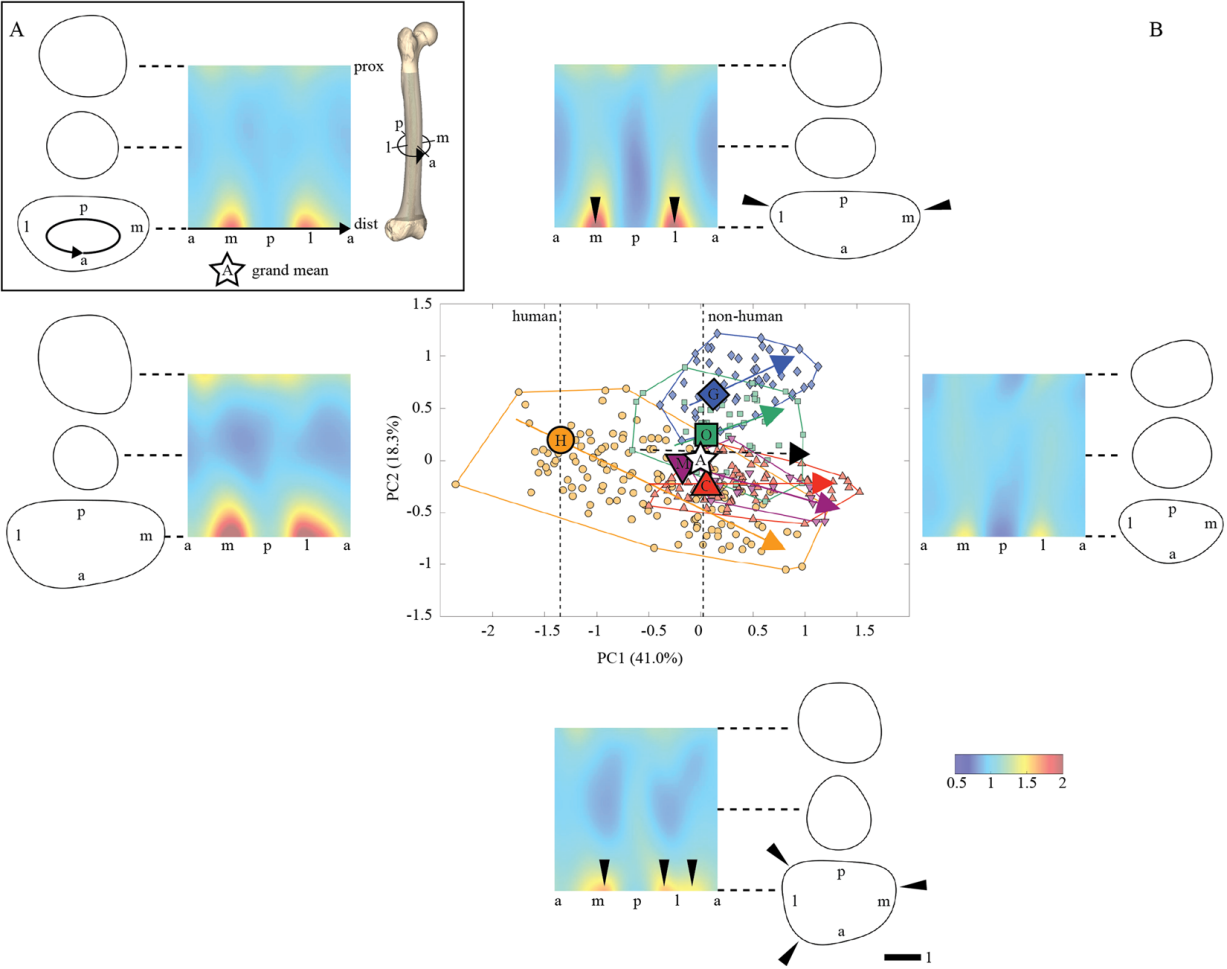
Femoral diaphyseal shape variation in ontogenetic series of humans, great apes, and Japanese macaques. (**A**) Scheme of morphometric mapping and a map corresponding to the grand mean diaphyseal shape (a-m-p-l: anterior-medial-posterior-lateral; the black arrow indicates the direction along which the diaphysis is “unrolled”). (**B**) Diaphyseal shape variation in PC space (along PC1 and PC2), and in physical space (morphometric maps and diaphyseal cross sections). Symbols used in the PC graph: filled circles: humans, upward triangles: chimpanzees, diamonds: gorillas, squares: orangutans, downward triangles: Japanese macaques; the large markers indicate the location of neonates; lines with arrowheads indicate taxon-specific ontogenetic trajectories; note offset of the human trajectory along −PC1, relative to great ape and macaque trajectories. The black dashed line with arrowhead indicates common ontogenetic trajectory. The morphometric maps and associated diaphyseal cross sections visualize shape variation along PC1 and PC2 (false-color code indicates external radius *r*_ext_). Major patterns of variation are as follows: PC1: (−) large proximal and distal diaphysis, (+) uniform diameter along the diaphysis. The distal metaphysis shows two peaks of *r*_ext_, corresponding to the mediolaterally wide distal cross section (arrowheads). PC2: (−) anteroposteriorly increased diameter of diaphysis, (+) mediolaterally increased diameter of diaphysis. PC2 (+): mediolaterally increased diameter of diaphysis. The distal metaphysis shows three peaks of *r*_ext_ (arrowheads).

The morphometric data obtained for each diaphysis (i.e., all *r*_ext_ values) were normalized to their respective median value, and mapped onto a cylindrical coordinate system (*ρ*, *θ*, *z*), where *ρ* = 1/(2π) = const. denotes the radius of the cylinder, angle *θ* denotes the anatomical direction (*θ* = 0° → 360°: anterior → medial → posterior → lateral → anterior; thus the diaphysis is “unrolled” along this direction), and *z* denotes the length-normalized position along the diaphysis (*z* = 0 → 1: distal → proximal)^37,38^. Since *ρ* = const., data can be visualized as two-dimensional morphometric maps **M**(*θ*, *z*), and distributions *r*_*ext*_(*θ*, *z*) (Fig. 1A) can be represented as *K* × *L* matrices, where *K* and *L* denote the number of elements along *z* and *θ*, respectively (*K* = *L* = 300). Note that this approach is analogous to dense sampling of morphometric data by using semi-landmarks on the diaphyseal surface. In both cases, independent normalization of the radial and length dimensions of the diaphysis yields two independent size measurements: median diaphyseal radius (*r*_d_) and diaphyseal length (*l*_d_)^36,41^. Thus the workflow of our method is analogous to that of conventional landmark-based geometric morphometrics.

For the comparative analysis of the morphometric maps **M**, 2D-Fourier transforms *F*(**M**) were calculated (**M** has a natural periodicity in *θ*), yielding *K* × *L* sets of Fourier coefficients. Note that the Fourier transform is applied to the size-corrected data. Specimens were aligned to each other by minimizing inter-specimen distances in Fourier space through rotation around *θ* (diaphyseal axis). To identify principal patterns of shape variation in the sample, Fourier coefficient sets were subjected to Principal Components Analysis (PCA). This procedure maps any specific diaphyseal morphology onto one specific point in PC space. To facilitate visual inspection and anatomical interpretation of the results of PCA, real-space morphometric maps were reconstructed by transforming a given point P* in PC space into its corresponding set of Fourier coefficients *F*(M*), and applying an inverse Fourier transformation to obtain a morphometric map M*. Morphometric maps were false-color coded. Diaphyseal length *l*_d_ is an indicator of the developmental stage^17,26^. Regression of **M** (diaphyseal shape) on length *l*_d_ thus yields taxon-specific allometric trajectories of ontogenetic shape change. In our case, these ontogenetic trajectories are approximately linear, such that they can be quantified with multivariate linear regression, resulting in taxon-specific ontogenetic shape vectors in PC space^42–44^. Taxon-specific trajectories are characterized by their direction through PC space, their length, and their location. The direction is specified by the unit allometric shape vector *a*, the length by the data variance along this vector, and the location by the centroid of taxon-specific distributions. Furthermore, for each taxon, the onset of postnatal development was determined by projecting the position of neonate specimens with known age (see Table S1) onto the allometric shape vector.

**Figure 2:**
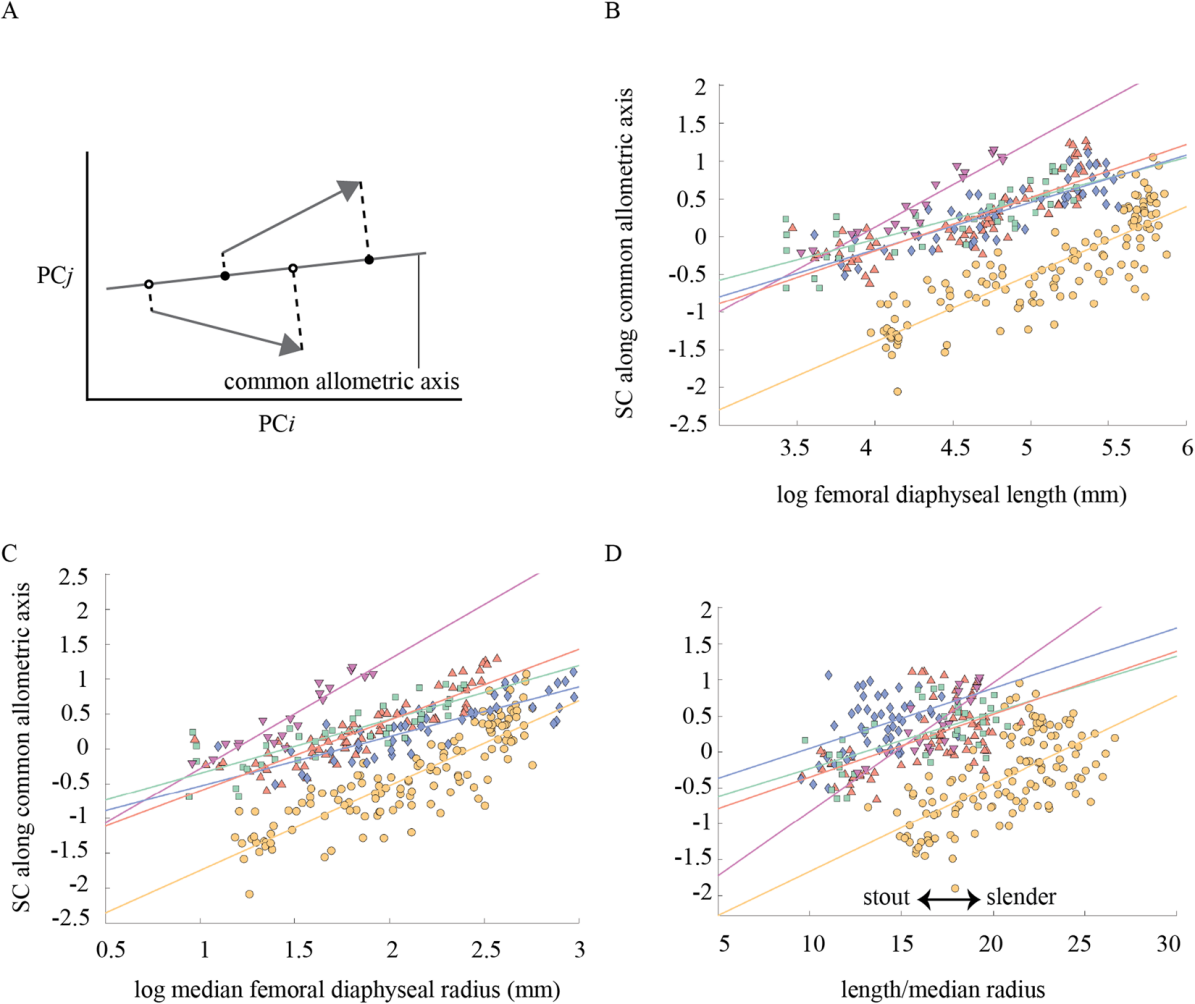
Ontogeny of diaphyseal shape as a function of length and radius (symbols as in Fig. 1). (**A**) Calculation of common allometric shape scores, SC. SC plotted against log femoral diaphyseal length *l*_d_ (**B**), log median femoral diaphyseal radius *r*_d_ (**C**), and ratio of length to median radius, *l*_d_/*r*_d_, as a measure of femoral robusticity (**D**). All graphs show a substantial offset of the human ontogenetic trajectory relative to non-human trajectories.

Using these data, differences between taxon-specific trajectories can be quantified as differences in direction, length and location. Differences in direction are expressed as trajectory divergence *V*_*ij*_

$${V}_{ij}=1-{({a}_{i}\cdot {a}_{j})}^{2},$$

where **a**_*i*_ and **a**_*j*_ are unit trajectory direction vectors of taxa *i* and *j*. Larger values of *V*_*ij*_ indicate larger divergence (*Vij* = 0 and *Vij* = 1 indicate parallelism and orthogonality of two vectors, respectively). Typically, the divergence between taxon-specific trajectories is moderate. It is thus sensible to calculate the average vector of all taxon-specific trajectory vectors, which captures a pattern of ontogenetic shape change shared by all taxa (Figs 1B, 2A). Projecting each specimen onto this common trajectory, yields the ontogenetic shape scores used in Fig. 2B–D. Statistical tests on differences of ontogenetic trajectory direction and position between groups were performed with bootstrapping (1,000 resamplings). All calculations were performed using the in-house program ForMATit developed by NM based on MATLAB 9.0 (MathWorks) (see ref.^36^ for further details).

Overall, thus, morphometric maps **M** quantify the morphology of the femoral diaphysis with reference to the shape of a Euclidean cylinder. Each point on the morphometric map indicates the local deviation of the diaphysis from the corresponding point on the cylinder, or alternatively, the amount of local deformation required to transform the cylinder into the specific diaphyseal morphology. Furthermore, the size of each diaphysis is measured by two quantities, diaphyseal length *l*_d_ and the median diaphyseal radius *r*_d_. Together with these size measurements, the morphometric maps fully specify diaphyseal morphology and permit assessment of various structurally and biomechanically relevant quantities, for example overall femoral stoutness (radius to length), and local cross-sectional properties such as platymery and second moments of inertia (bending moments)^36^. Furthermore, the application to morphometric maps of PCA as a standard multivariate technique permits evaluation and visualization of taxon- and/or stage-specific mean diaphyseal shapes, and of taxon-specific ontogenetic trajectories of diaphyseal shape change. It is important to keep in mind that PC axes are convenient statistical constructs for the visualization of multivariate data in low-dimensional spaces, but that – *per se* – they do not necessarily express biologically relevant patterns of shape variation.

## Results

Figures 1 and S2 show diaphyseal shape variation of the sample in PC space, and corresponding patterns of shape variation on morphometric maps. PCs 1−3 comprise 41.0%, 18.3%, and 7.7% of the total variance, respectively, and reveal taxon-specific structuring of the data. The higher-order PCs, which together account for the remaining 33% of variation, do not reveal further structuring and are thus not visualized here. Taxa differ in diaphyseal shape (as expressed by the different locations of taxon-specific data in PC space) as well as in patterns of diaphyseal shape change during ontogeny (as expressed by different lengths and/or directions of taxon-specific ontogenetic trajectory vectors). All trajectory vectors have their main orientation along PC1, such that PC1 largely captures a pattern of ontogenetic shape change shared by all taxa used in this study.

The principal pattern captured by PC1 is variation in relative width of the metaphyseal regions. Younger individuals (*i.e*., lower PC1 scores) have diaphyses with relatively large metaphyses. The distal metaphysis is mediolaterally wide, while the proximal metaphysis has a more rounded shape. Older individuals (*i.e*., higher PC1 scores) have diaphyses with a more uniform radius along the entire length. Compared to great ape and macaque trajectories, the human trajectory exhibits a marked shift and elongation along –PC1. Taxon-specific trajectories diverge mostly along PC2 (Fig. 1B). PC2 largely reflects variation in diaphyseal platymery. Low PC2 scores (*e.g*. in adult chimpanzees, Japanese macaques, and humans) correspond to a rounded diaphysis and distal metaphysis. High PC2 scores (*e.g*. in adult gorillas and orangutans) correspond to a more platymeric diaphysis. The human trajectory also diverges from all non-human trajectories along PC3 (Fig. S2). PC3 reflects variation in relative size of proximal metaphysis and of shape of distal metaphysis. Higher PC3 scores (adult humans) correspond to large and mediolaterally expanded distal relative to proximal metaphyses. The distal metaphysis corresponding to high PC3 score also exhibits a developed anterolateral ridge (lateral patellar lip).

Table 1 shows that humans exhibit significantly longer ontogenetic trajectories than great apes and Japanese macaques. Table 2 shows that chimpanzees are morphologically as distant from gorillas as from humans. Table 3 shows that the direction of the human trajectory differs from that of the great apes (but not from Japanese macaques; tested by resampling). Furthermore, the directions of the gorilla and orangutan trajectories do not differ significantly from each other, although they both differ from the chimpanzee trajectory. Overall, the trajectory of chimpanzees is closer to that of humans and Japanese macaques than to that of gorillas in terms of trajectory position and direction.

**Table 1:** Variance along taxon-specific ontogenetic trajectories, and *p*-values of F-test on between-group differences in variance.

Taxon (taxon-specific variance)	Humans	Chimps	Gorillas	Orangutans
Human (0.57)	—	—	—	—
Chimps (0.24)	<0.01	—	—	—
Gorillas (0.16)	<0.01	0.17	—	—
Orangutans (0.16)	<0.01	0.21	0.99	—
Macaques (0.23)	<0.05	0.95	0.35	0.37

**Table 2:** Distances between mean points of ontogenetic trajectories.

	Humans	Chimps	Gorillas	Orangutans
Chimps	0.91*	—	—	—
Gorillas	1.29*	0.96*	−	—
Orangutans	0.94*	0.61*	0.41*	—
Macaques	1.02*	0.21	0.98*	0.63*

*p < 0.05.

**Table 3:** Divergence of ontogenetic trajectory vectors.

	Humans	Chimps	Gorillas	Orangutans
Chimps	0.28*	−	−	−
Gorillas	0.63*	0.32*	−	−
Orangutans	0.35*	0.22*	0.09	−
Macaques	0.07	0.10	0.54	0.31

*p < 0.05.

Figure 2 graphs the common component of diaphyseal shape change (common allometric ontogenetic scores^42^), which coincides with PC1. Humans show significantly lower scores at a given diaphyseal length (Fig. 2B), diaphyseal radius (Fig. 2C), and robustness index (the ratio of length to radius) (Fig. 2D) [the regression line of humans is located significantly lower than those of great apes and Japanese macaques (*p* < 0.01; tested by Quick Test^45^). Specifically, human neonates exhibit substantially lower PC1 scores than the neonates of great apes and Japanese macaques. Conversely, the PC1 scores of neonate great apes and Japanese macaques are comparable to the scores of humans at later ontogenetic stages. The slope in Fig. 2B represents the amount of shape change per unit diaphyseal length. The great apes show significantly shallower slopes than humans and Japanese macaques (*p* < 0.01). The values of the slopes were not statistically different between humans and Japanese macaques.

**Figure 3:**
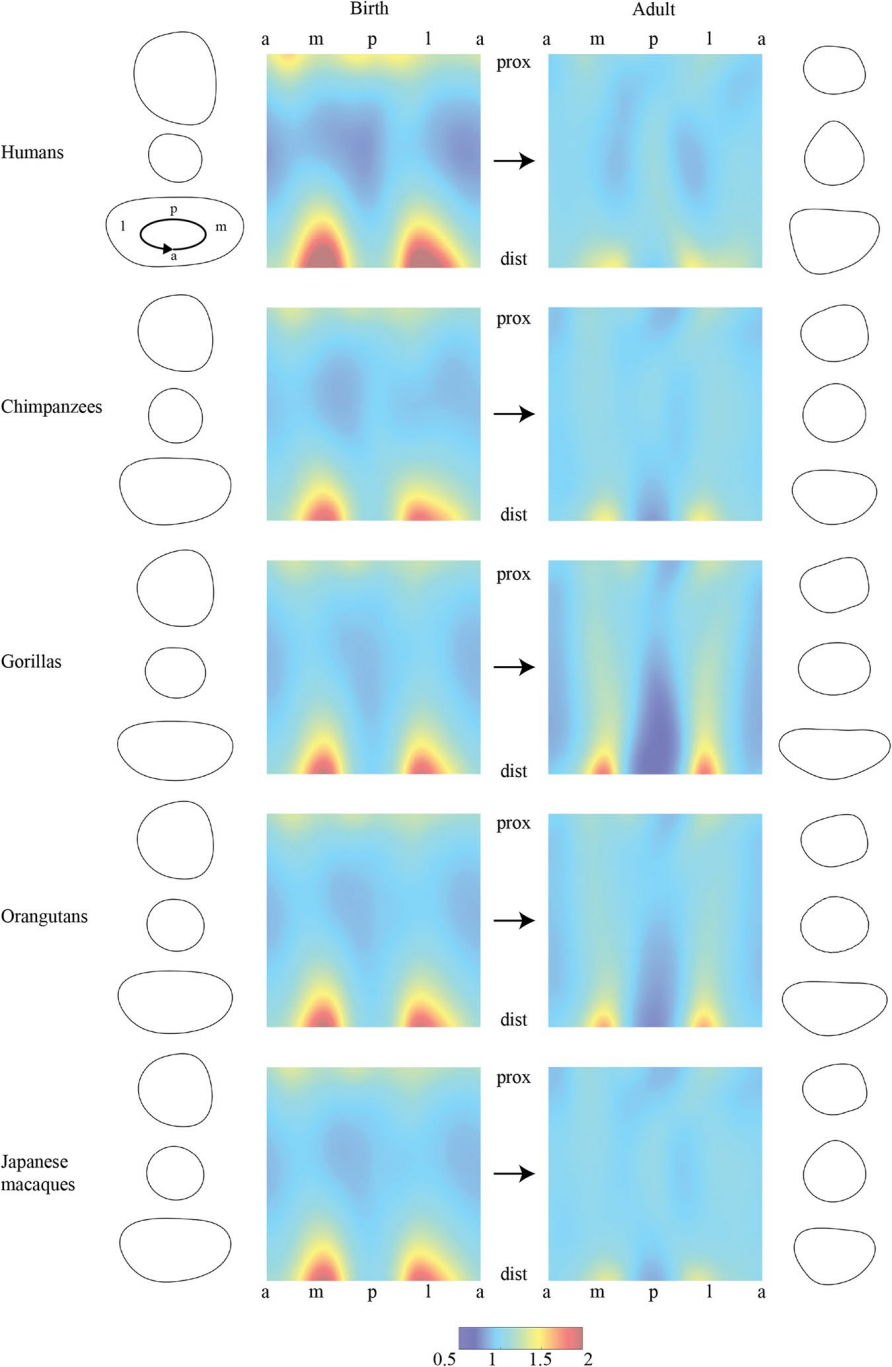
Taxon-specific ontogeny of the femoral diaphyseal morphology (corresponding to the ontogenetic trajectories indicated in Fig. 1). In all taxa, the metaphyseal regions are relatively large compared to the middle diaphysis at birth than at adult stage. In human neonates, proximal and distal metaphyses exhibit relatively larger mediolateral diameters compared to the non-human taxa. Independent of ontogenetic stage, humans, chimpanzees, and Japanese macaques exhibit relatively rounded diaphyseal morphologies whereas gorillas and orangutans exhibit more platymeric morphologies indicated by the red-colored stripes along medial and lateral directions.

Figure 3 summarizes commonalities and differences between taxon-specific patterns of femoral diaphyseal development. Since the ontogenetic trajectories of all taxa examined here are largely linear, we visualize neonate and adult morphologies as start and end points, respectively. The morphometric maps and cross-sectional outlines show that taxon-specific diaphyseal features are already present at birth. During postnatal ontogeny, all taxa exhibit a shared pattern of expansion of the proximal and distal metaphyses. Patterns of diaphyseal ontogenetic shape change differ conspicuously between chimpanzees and gorillas. Furthermore, the diaphysis is fairly rounded in chimpanzees and Japanese macaques, while it is relatively anteroposteriorly and mediolaterally expanded in humans and in gorillas/orangutans.

## Discussion

The results show that the femoral diaphyses of chimpanzees and gorillas exhibit strikingly divergent ontogenetic trajectories. The femoral diaphyseal ontogeny of chimpanzees is as different from that of gorillas as it is from that of humans and Japanese macaques (Fig. 1). Orangutans – rather than chimpanzees – are closest to gorillas at any stage of ontogeny. The major differences between chimpanzees and gorillas are found along PC2 (Fig. 1). Taxon-specific differences between adult chimpanzees and gorillas along PC2 cannot be explained by differences in adult body size (taxon-specific mean PC2 values of adult specimens vs. reported mean body weights^46,47^; R^2^ = 0.49, *p* = 0.19; see Supplementary Note 1, Fig. S3 and Table S2 for regression analysis of PC2 scores vs. body mass).

There are two possible hypotheses for explaining the disparities of femoral ontogenetic patterns observed between chimpanzees and gorillas. The first is that these disparities largely reflect taxon-specific developmental programs, and that the role of *in vivo* bone modification is relatively small. Our data show that chimpanzees and gorillas follow different patterns of diaphyseal shape change, despite similar locomotor behaviors. Interestingly, orangutans, which exhibit substantially different locomotor behaviors from gorillas, show a similar ontogenetic pattern of femoral diaphysis. Together, these data support the hypothesis that the taxon-specific ontogenetic patterns of the femoral diaphysis observed here do not reflect taxon-specific *in vivo* locomotor behaviors. This is consistent with various experimental and morphometric studies showing that taxon-specific differences in *in vivo* activity patterns have relatively little influence on the development of postcranial skeletal morphology^36,48,49^. The second hypothesis is that chimpanzees and gorillas engage in different modes of knuckle walking on terrestrial substrates as proposed by Kivell and Schmitt^22^. It is possible that chimpanzees and gorillas use the hind limbs for knuckle walking in different ways, as indicated by different hind limb muscular allocation^50,51^ and different patterns of muscular attachment on the femur^15^. Data pertaining to how hind limb usage differs in chimpanzees and gorillas are still scarce, however, and it is thus difficult to test this hypothesis.

The two hypotheses are not mutually exclusive, such that it remains unknown to which extent femoral ontogeny reflects taxon-specific developmental programs versus actual taxon-specific differences in knuckle walking. In either case, our data show that chimpanzees and gorillas do not have a shared pattern of postnatal diaphyseal ontogeny, despite similar locomotor behaviors. Furthermore, the femoral morphologies of chimpanzees and gorillas are already distinct around birth^19^, indicating that their ontogenetic programs diverged already before birth, *i.e*., independent of locomotor activity. Overall, thus, our data indicate that chimpanzees and gorillas each represent derived patterns of femoral diaphyseal ontogeny, rather than a conserved GCH-LCA pattern. This is consistent with the “generalized quadruped ancestor” hypothesis, and suggests that these species cannot be used as a model for the musculoskeletal structure of the LCA of African great apes and humans^8,11,52^.

In addition to the chimpanzee−gorilla disparity, our data also show a complex relationship between form and function in the femoral diaphysis. It has been proposed that a rounded shape of the diaphysis is suitable for arboreal locomotion, since bending strength is uniformly distributed around the diaphysis^53,54^. Our data show that the degree of arboreality is not necessarily correlated with the degree of diaphyseal cross-sectional roundness in the hominids. Gorillas, which are mostly terrestrial (especially males^55,56^), and orangutans, which are mostly arboreal, exhibit relatively platymeric (mediolaterally expanded) diaphyses compared to macaques, chimpanzees, and humans (Fig. 1). This indicates that the degree of arboreality is not the only factor influencing diaphyseal platymery. One additional factor would be the structure of the muscles around the femoral diaphysis^14,15^. For example, Suwa *et al*. (2012) proposed that structural similarities of the proximal femur of gorillas and orangutans could reflect increased volume of the adductor/hamstring complex in gorillas, and increased mobility of the hip joints in orangutans^57,58^. Interpreting the degree of platymery of long bone diaphyses in terms of locomotor behaviors should thus be done with caution.

Our results further indicates that – apart from taxon-specific differences – there exists a basic pattern of femoral diaphyseal ontogeny that is shared by humans, chimpanzees, gorillas, orangutans, and even Japanese macaques, and that independent of taxon-specific locomotor behaviors and body size. We hypothesize that the evolutionary origin of this pattern dates back to the last common ancestor of the taxa studied here, *i.e*. to at least 30 mya^59^. To test this hypothesis more formally, however, an expanded sample representing a wider range of primate taxa would be needed. The morphometric mapping-based analyses show that the proximal and distal metaphyses of the diaphysis are relatively large and mediolaterally wide during early ontogeny, becoming smaller as the animal approaches adulthood. This is consistent with earlier observations on the femoral ontogeny in chimpanzees^60^. Interestingly, an experimental study on mice showed that the expression levels of growth factors such as IGF-IR (insulin-like growth factor-I) and PCNA (proliferating cell nuclear antigen) decrease at the proximal epiphysis, but remain relatively high at the distal epiphysis of the femur, and that the expression level is associated with the size of the growth plate^61^. Since epiphyseal and metaphyseal sizes are correlated, these findings can be paralleled with the results presented here showing that the distal femoral metaphyseal region remains relatively larger than the proximal metaphyseal region (Fig. 1). We thus hypothesize that there is a link between these developmental processes at the molecular level and the developmental patterns at the macro-morphological level observed in this study. While this hypothesis remains to be tested, it proposes a link between evolutionary changes in growth factor expression and the evolutionary diversification of primate femoral ontogeny and morphology.

Humans exhibit a pattern of femoral diaphyseal ontogeny that is markedly distinct from that of the non-human taxa studied here (Figs 1 and 3). Compared to great apes and macaques, the onset of the human postnatal ontogenetic trajectory is shifted “backwards” in PC space (*i.e*., toward juvenile forms). Overall, however, the human trajectory is longer, such that adult humans are at similar positions along PC1 as adults of the non-human taxa analyzed here. This results in a greater degree of femoral shape change in humans compared to the other taxa (Fig. 1, Table 1). How are these differences related to differences in skeletal growth and life history? In humans and great apes, the duration of prenatal ontogeny (*i.e*., gestation time) is largely comparable^62^. However, postnatal somatic growth (as measured by increase in stature and body mass) is completed around 20y and 10y, respectively^62,63^, while estimates for the maximum lifespan are 85y and 55y, respectively^64,65^. Humans thus require more time to complete somatic growth, both in absolute terms, and relative to the duration of the prenatal and of the postnatal lifespan. It remains to be investigated how the human-specific pattern of femoral development is related to secondary altriciality and a generally slow life history. Compared to the femoral diaphyseal morphologies of great ape neonates, human neonates indeed exhibit “underdeveloped” diaphyseal morphologies (Fig. 1). On the other hand, we hypothesize that the human-specific pattern of postnatal femoral development results from a combination of higher growth activity during early postnatal life in association with an extended period of growth. Overall, our data indicate that the human-specific program of femoral diaphyseal ontogeny was brought about by retardation, rather than acceleration, of the developmental program when compared with great apes. It is sensible to assume that these modifications evolved in association with femoral elongation, and the acquisition of committed bipedality with an upright posture. Such a shift in developmental programs could have occurred with the emergence of the australopithecines^35^. Our data further show that the human-specific femoral diaphyseal morphology is already present before birth, thus before the onset of locomotion. This indicates that human fetal development underwent substantial evolutionary modifications^19^. Data pertaining to prenatal long bone development of great apes are scarce, such that direct quantitative comparisons are currently difficult. In any case, the fact that taxon-specific differences are already present at birth indicates a strong genetic determinant of femoral diaphyseal morphology. While we focused on differences between humans and great apes in this study, it should be noted that femoral morphology (*e.g*., robusticity) exhibits considerable variation in modern *H. sapiens*, early *H. sapiens*, *H. neanderthalensis* and *H. erectus*^39,66–70^, such that it is likely that diversification of the femoral development should have also occurred in our genus *Homo*.

Due to the disparity between patterns of phylogenetic, locomotor and femoral morphological diversification of the great apes, it is difficult to infer the primitive state of femoral diaphyseal morphology and locomotor mode of the hominoids. Further research on fossils^13^, and examination of the variation in muscle structure and development^14,15,71,72^ across phenotypic features are needed to infer the state of the human–chimpanzee and/or human–chimpanzee–gorilla LCAs^73^, and the evolutionary context in which human bipedality emerged.

## Conclusion

This study compared ontogenetic patterns of femoral diaphyseal shape change among humans, great apes, and Japanese macaques. Chimpanzees and gorillas, both of which engage in a specific mode of terrestrial locomotion– knuckle walking−, exhibit distinct developmental patterns. This is consistent with the hypothesis that phenotypic variation in chimpanzees and gorillas reflects independent evolutionary histories in each taxon, rather than a common knuckle-walking ancestor. Humans exhibit a highly derived pattern of femoral development, exhibiting differential elongation of the femur associated with the evolution of committed bipedality. Apart from taxon-specific differences, humans, great apes, and Japanese macaques share a basic pattern of the femoral diaphyseal development, indicating deep evolutionary roots of the underlying developmental program. Overall, our study shows that taxon-specific femoral diaphyseal morphologies reflect a combination of phyletic history, basic developmental constraints, and taxon-specific adaptations.

## Electronic supplementary material

Supplementary Information
