# Supplementary material for: Femoral ontogeny in humans and great apes and its implications for their last common ancestor
Source: Sci Rep. 2018 Jan 31;8:1930. doi: 10.1038/s41598-018-20410-4 (PMC5792642; doi:10.1038/s41598-018-20410-4)
Supplement: Supplementary file 1 — Supplementary Information [file 41598_2018_20410_MOESM1_ESM.doc]

Supplementary Information

Femoral ontogeny in humans and great apes and its implications for their last common ancestor

Naoki Morimoto^1*^, Masato Nakatsukasa^1^, Marcia S. Ponce de León^2^, Christoph P.E. Zollikofer^2^

^1^ *Laboratory of Physical Anthropology, Graduate School of Science, Kyoto University, Japan*

^2^ Anthropological Institute and Museum, University of Zurich, Switzerland

^*^Correspondence to N.M. ([morimoto@anthro.zool.kyoto-u.ac.jp](mailto:morimoto@anthro.zool.kyoto-u.ac.jp))

**Supplementary Note 1**

We also tested whether PC2 scores are correlated with body mass for each taxon along ontogeny. Since body mass is not measureable for every specimen used in this study, we used the mean cross-sectional area of the femoral diaphysis as a proxy (calculated as the mean value of all cross-sections along the entire length of femoral diaphysis). The results show that the PC2 scores are positively correlated with diaphyseal cross-sectional area in gorillas and orangutans, while they are negatively correlated with the cross-sectional area in humans (and macaques) (Supplementary Fig. S3 and Table S2). Following the logic of geometric morphometrics^1^, different directions of the ontogenetic trajectories in morphospace correspond to different patterns of shape change in real space. In our dataset, while the mediolateral diameter of the femoral diaphysis increases relative to the anteroposterior diameter along ontogeny in gorillas, mediolateral and anteroposterior diameters remain fairly constant, relative to each other, in chimpanzees.

**Reference**

1 Bookstein, F*. Morphometric Tools for Landmark Data: Geometry and Biolo*gy. (Camnridge University Press, 1991).

**Supplementary Figures**


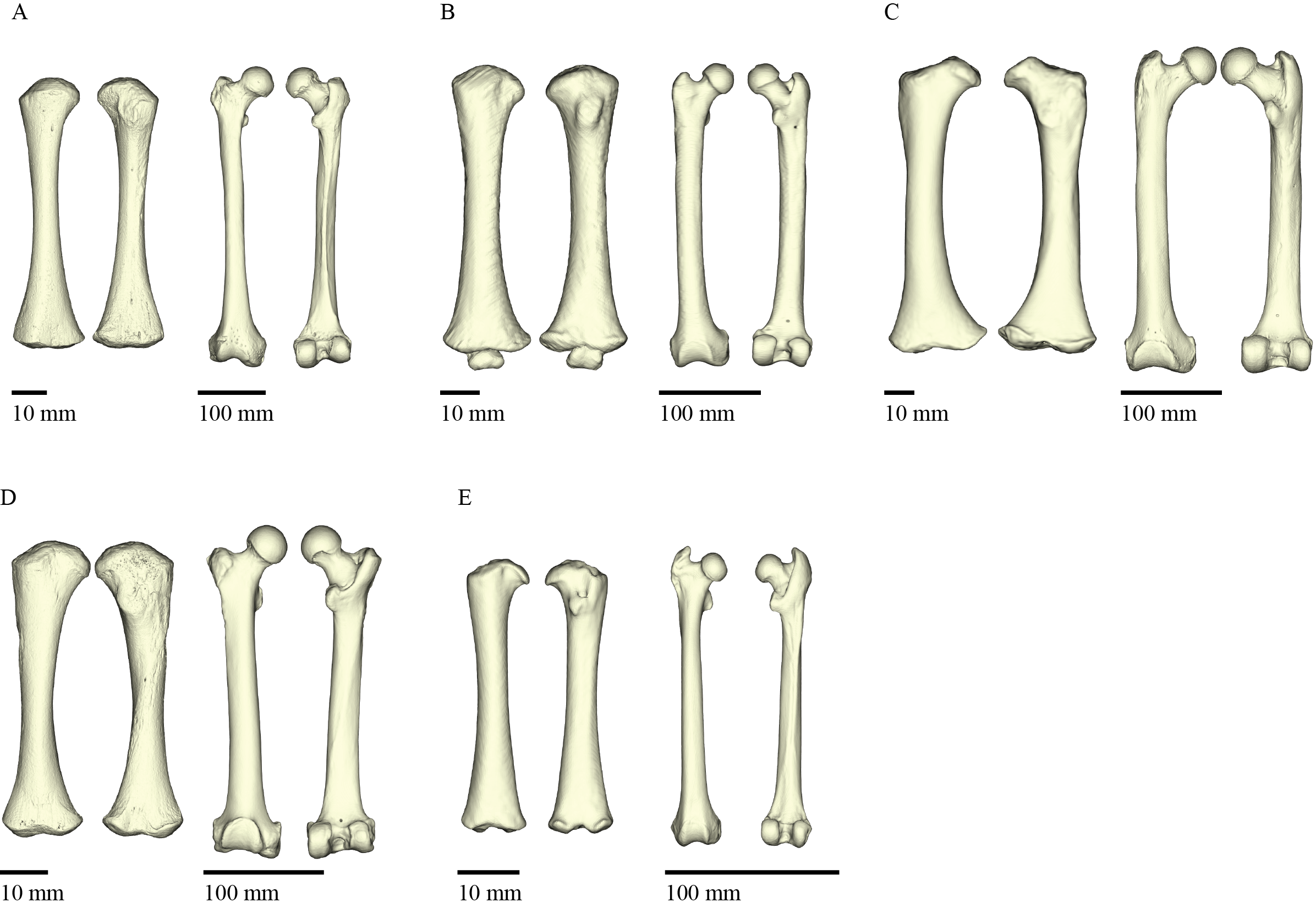


Fig. S1 The femur at neonatal (left) and adult (right) stages. Note that the epiphyses are not ossified in immature stage. A: *Homo sapiens*, B: *Pan troglodytes*, C: *Gorilla gorilla*, D: *Pongo pygmaeus*, E: *Macaca fuscata*.


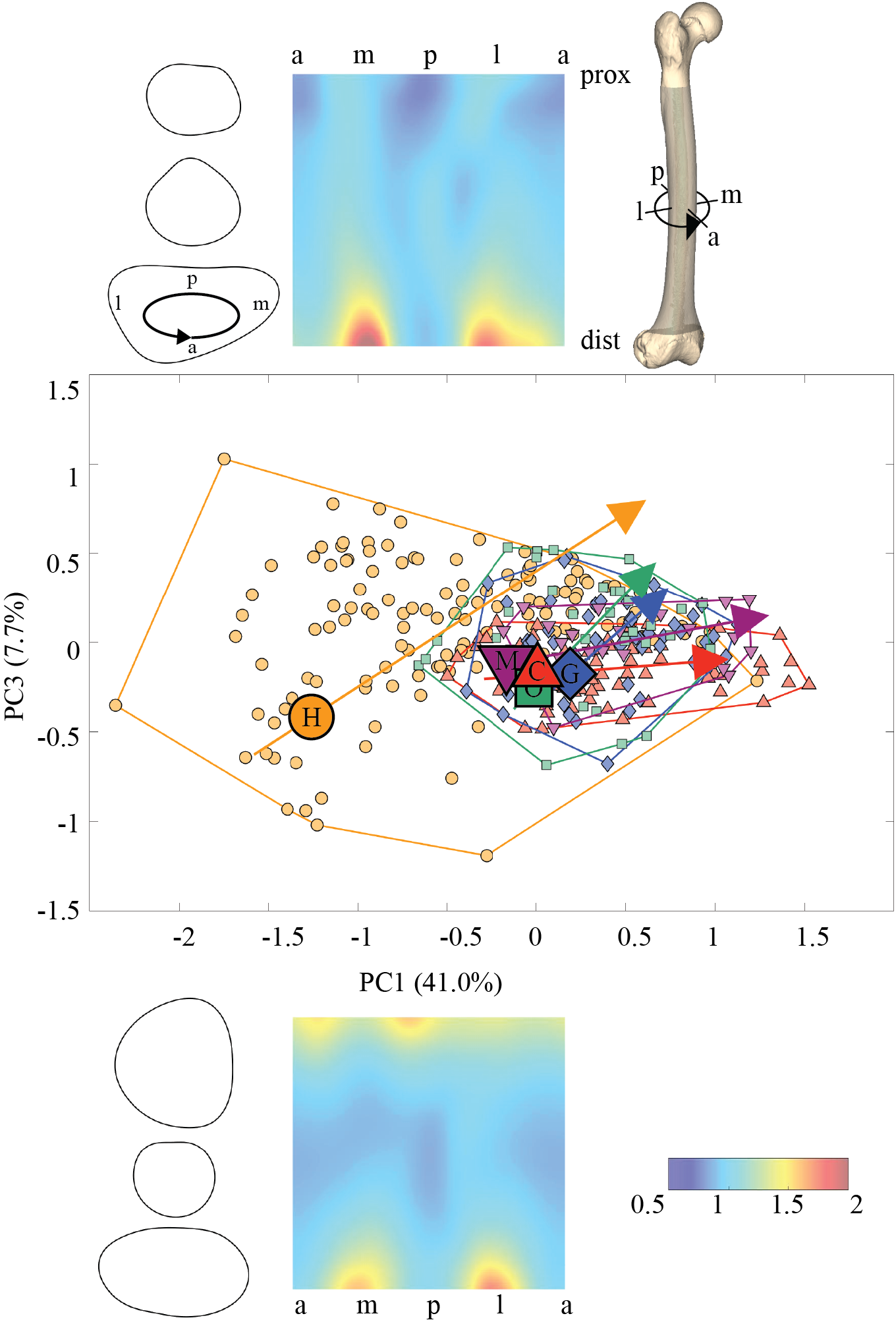


Fig. S2 Shape variation along PC3. Filled circles: humans, upward triangles: chimpanzees, diamonds: gorillas, squares: orangutans, downward triangles: Japanese macaques. The large markers indicate the neonate specimens with known age at death. The morphometric maps show that humans (PC3+) exhibit mediolaterally wider distal metaphysis relative to the proximal metaphysis.


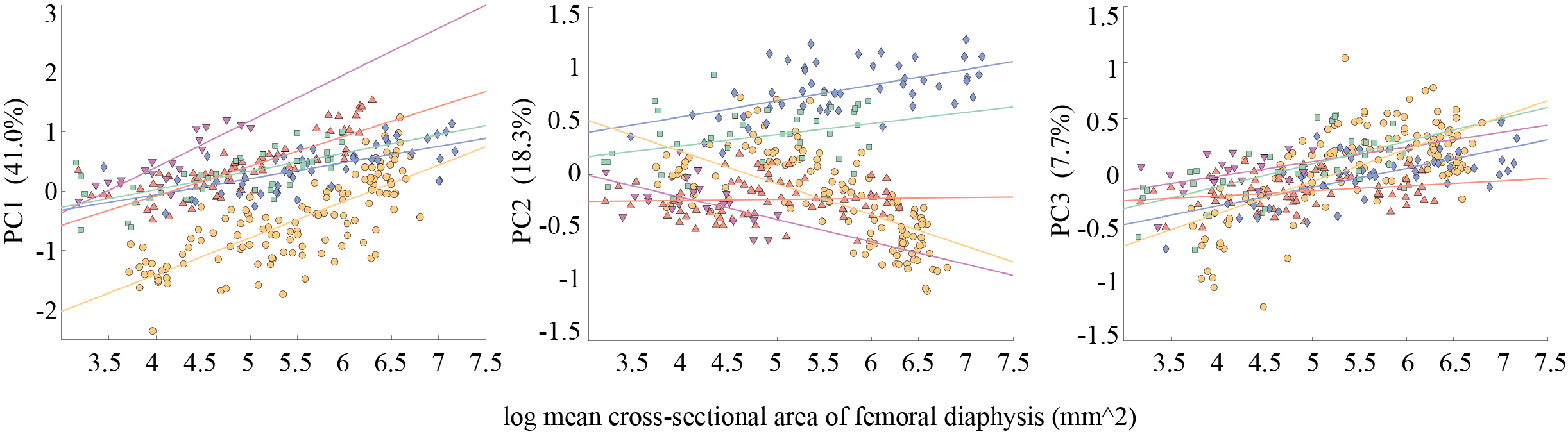


Fig. S3 Log mean cross-sectional area of femoral diaphysis vs. PC scores (PC1-3). Coefficients of least square fitting are shown in Supplementary Table S2. The PC1 and PC3 are mostly positively correlated with cross-sectional area of femoral diaphysis in all the taxa, while the patterns of correlation are different in PC2 between humans and Japanese macaques (negative correlation) and gorillas and orangutans (positive correlation).

**Supplementary Tables**

Table S1 Sample structure. Abbreviations: AIMUZH: Anthropological Institute and Museum, University of Zurich ("Anonymous" indicates forensic individuals obtained from the digital autopsy database of the Catholic University of Leuven, Belgium). CARC: Congenital

Anomaly Research Center of Kyoto University, Kyoto, Japan. PRI: Primate Research Institute of Kyoto University, Japan. MRAC: Royal Africa Museum, Tervuren, Belgium.

Notes: ^1^documented age at death: 40 gestational weeks, ^2^downloaded from <http://dmm3.pri.kyoto-u.ac.jp/dmm/WebGallery/index.html>, ^3^documented age at death: 2 minutes, ^4^documented age at death: 0 minutes, ^5^documented age at death: 3 days

| ID | sex | Femoral diaphyseal length (mm) | population | wild/captive | notes |
| --- | --- | --- | --- | --- | --- |
| CARC38630 | f | 58.8 | Modern Japanese | - | 1 |
| AIMUZH HF 1263-2 | - | 53.2 | Swiss Medieval | - |  |
| AIMUZH HF 1274-2-2 | - | 56.6 | Swiss Medieval | - |  |
| AIMUZH11184 | - | 57.7 | Swiss Medieval | - |  |
| AIMUZH HF 1277-1a-1-1 | - | 58.6 | Swiss Medieval | - |  |
| AIMUZH HF 1269A-1-1 | - | 59.0 | Swiss Medieval | - |  |
| AIMUZH HF 1277-1c-1 | - | 59.4 | Swiss Medieval | - |  |
| AIMUZH HF 1275-1 | - | 60.1 | Swiss Medieval | - |  |
| AIMUZH HF 1278-2-1 | - | 60.6 | Swiss Medieval | - |  |
| AIMUZH HF 1276-2-2 | - | 60.7 | Swiss Medieval | - |  |
| AIMUZH HF 1277-1a-1-8 | - | 60.8 | Swiss Medieval | - |  |
| AIMUZH HF 1277-1-a1-9 | - | 60.9 | Swiss Medieval | - |  |
| AIMUZH pk 0 Femur 3 | - | 61.5 | Swiss Medieval | - |  |
| AIMUZH T21 | - | 62.0 | Swiss Medieval | - |  |
| AIMUZH11163 | - | 62.7 | Swiss Medieval | - |  |
| AIMUZH HF 1267-1 | - | 63.0 | Swiss Medieval | - |  |
| AIMUZH HF 1277-1a-1-2 | - | 63.1 | Swiss Medieval | - |  |
| AIMUZH HF 1237-g-2 | - | 63.2 | Swiss Medieval | - |  |
| AIMUZH HF 1277-1a-1-7 | - | 63.4 | Swiss Medieval | - |  |
| AIMUZH HF 1277-1c-7 | - | 63.5 | Swiss Medieval | - |  |
| AIMUZH Anonymous 1 | f | 66.8 | Modern Europe | - |  |
| AIMUZH AS255 | - | 83.2 | Swiss Medieval | - |  |
| AIMUZH Anonymous 7 | m | 88.1 | Modern Europe | - |  |
| AIMUZH PHB-1 | - | 104.4 | Hungary Neolithic | - |  |
| AIMUZH Anonymous 2 | f | 147.2 | Modern Europe | - |  |
| AIMUZH Anonymous 3 | f | 172.3 | Modern Europe | - |  |
| AIMUZH Anonymous 4 | f | 186.2 | Modern Europe | - |  |
| AIMUZH Anonymous 5 | m | 201.3 | Modern Europe | - |  |
| AIMUZH Anonymous 6 | f | 337.1 | Modern Europe | - |  |
| AIMUZH pk 0 Femur 2 | - | 67.2 | Swiss Medieval | - |  |
| AIMUZH T150 | - | 76.5 | Swiss Medieval | - |  |
| AIMUZH T22 | - | 78.6 | Swiss Medieval | - |  |
| AIMUZH683 | - | 85.5 | Swiss Medieval | - |  |
| AIMUZH5713 | - | 85.9 | Swiss Medieval | - |  |
| AIMUZH654 | - | 86.6 | Swiss Medieval | - |  |
| AIMUZH pk 0 Femur 1 | - | 88.1 | Swiss Medieval | - |  |
| AIMUZH T19 | - | 88.5 | Swiss Medieval | - |  |
| AIMUZH arch Fr 2 | - | 99.6 | Swiss Medieval | - |  |
| AIMUZH T393 | - | 98.3 | Swiss Medieval | - |  |
| AIMUZH pk 0 Femur 4 | - | 104.7 | Swiss Medieval | - |  |
| AIMUZH pk 0 Femur 5 | - | 108.5 | Swiss Medieval | - |  |
| AIMUZH1229 | - | 111.8 | Swiss Medieval | - |  |
| AIMUZH pk 2 Femur 1 | - | 111.3 | Swiss Medieval | - |  |
| AIMUZH pk 1 Femur 4 | - | 114.5 | Swiss Medieval | - |  |
| AIMUZH pk 2 Femur 4 | - | 116.5 | Swiss Medieval | - |  |
| AIMUZH pk 2 Femur 2 | - | 118.6 | Swiss Medieval | - |  |
| AIMUZH pk 3 Femur 1 | - | 120.2 | Swiss Medieval | - |  |
| AIMUZH pk 3 Femur 4 | - | 122.1 | Swiss Medieval | - |  |
| AIMUZH T402 | - | 123.6 | Swiss Medieval | - |  |
| AIMUZH1231 | - | 123.6 | Laas | - |  |
| AIMUZH pk 2 Femur 3 | - | 126.1 | Swiss Medieval | - |  |
| AIMUZH T243 | - | 138.3 | Swiss Medieval | - |  |
| AIMUZH15420 | - | 136.7 | Swiss Medieval | - |  |
| AIMUZH pk 3 Femur 2 | - | 138.5 | Swiss Medieval | - |  |
| AIMUZH T232 | - | 146.0 | Swiss Medieval | - |  |
| AIMUZH pk 4 Femur 3 | - | 149.7 | Swiss Medieval | - |  |
| AIMUZH pk 4 Femur 1 | - | 151.4 | Swiss Medieval | - |  |
| AIMUZH T96 | - | 151.2 | Swiss Medieval | - |  |
| AIMUZH T25 | - | 152.5 | Swiss Medieval | - |  |
| AIMUZH pk 4 Femur 2 | - | 158.0 | Swiss Medieval | - |  |
| AIMUZH1238 | - | 160.0 | Swiss Medieval | - |  |
| AIMUZH Grab 1-3676 | - | 171.0 | Swiss Medieval | - |  |
| AIMUZH789 Fl-1 | - | 173.0 | Laas | - |  |
| AIMUZH T384 | - | 173.0 | Swiss Medieval | - |  |
| AIMUZH pk 4 Femur 4 | - | 174.1 | Swiss Medieval | - |  |
| AIMUZH T418 | - | 174.8 | Swiss Medieval | - |  |
| AIMUZH T86 | - | 180.0 | Swiss Medieval | - |  |
| AIMUZH pk 6 | - | 180.2 | Swiss Medieval | - |  |
| AIMUZH1232 | - | 194.0 | Laas | - |  |
| AIMUZH942 | - | 195.0 | Laas | - |  |
| AIMUZH1237 | - | 197.5 | Laas | - |  |
| AIMUZH T188 | - | 203.0 | Swiss Medieval | - |  |
| AIMUZH T304 | - | 211.3 | Swiss Medieval | - |  |
| AIMUZH Grab 9 | - | 214.5 | Swiss Medieval | - |  |
| AIMUZH1236 | - | 223.0 | Laas | - |  |
| AIMUZH T57 | - | 223.0 | Swiss Medieval | - |  |
| AIMUZH T629 | - | 229.0 | Swiss Medieval | - |  |
| AIMUZH T240 | - | 230.0 | Swiss Medieval | - |  |
| AIMUZH T173 | - | 236.0 | Swiss Medieval | - |  |
| AIMUZH T242 | - | 237.0 | Swiss Medieval | - |  |
| AIMUZH T56 | - | 239.0 | Swiss Medieval | - |  |
| AIMUZH T94 | - | 247.0 | Swiss Medieval | - |  |
| AIMUZH943 | - | 256.2 | Laas | - |  |
| AIMUZH T244 | - | 266.0 | Swiss Medieval | - |  |
| AIMUZH1240 | - | 269.2 | Laas | - |  |
| AIMUZH789 | - | 274.5 | Laas | - |  |
| AIMUZH T581 | - | 276.0 | Swiss Medieval | - |  |
| AIMUZH T348 | - | 278.0 | Swiss Medieval | - |  |
| AIMUZH T234 | - | 279.0 | Swiss Medieval | - |  |
| AIMUZH372 | - | 289.3 | African | - |  |
| AIMUZH T404 | - | 290.0 | Swiss Medieval | - |  |
| AIMUZH T29 | - | 296.0 | Swiss Medieval | - |  |
| AIMUZH T413 | - | 296.2 | Swiss Medieval | - |  |
| AIMUZH T51 | - | 301.0 | Swiss Medieval | - |  |
| AIMUZH T170 | - | 306.0 | Swiss Medieval | - |  |
| AIMUZH T26 | - | 308.0 | Swiss Medieval | - |  |
| AIMUZH T195 | - | 310.0 | Swiss Medieval | - |  |
| AIMUZH T99 | - | 313.0 | Swiss Medieval | - |  |
| AIMUZH Grab 7-7 | - | 237.7 | Swiss Neolithic | - |  |
| AIMUZH Grab 14-16 | - | 308.0 | Swiss Neolithic | - |  |
| AIMUZH Grab 25-3 | - | 291.8 | Swiss Neolithic | - |  |
| AIMUZH Grab 10-10 | - | 318.6 | Swiss Neolithic | - |  |
| AIMUZH Grab 34-44 | - | 306.3 | Swiss Neolithic | - |  |
| AIMUZH Grab 59-15 | - | 321.4 | Swiss Neolithic | - |  |
| AIMUZH Grab 58-14 | - | 315.7 | Swiss Neolithic | - |  |
| AIMUZH Grab 90-23 | - | 322.6 | Swiss Neolithic | - |  |
| AIMUZH Grab 217 | - | 294.9 | Samtavro | - |  |
| AIMUZH Grab 276 | - | 337.7 | Samtavro | - |  |
| AIMUZH Grab 342 | - | 196.0 | Nokalaqevi | - |  |
| AIMUZH Grab 392 | - | 279.0 | Nokalaqevi | - |  |
| AIMUZH Grab 338 | - | 290.7 | Nokalaqevi | - |  |
| AIMUZH Grab 117 | - | 306.4 | Nokalaqevi | - |  |
| AIMUZH Grab 386 | - | 308.8 | Nokalaqevi | - |  |
| AIMUZH Grab 336 | - | 319.5 | Nokalaqevi | - |  |
| AIMUZH Grab 114 | - | 331.4 | Nokalaqevi | - |  |
| AIMUZH256 | f | 284.3 | Maori | - |  |
| AIMUZH257 | f | 290.7 | Maori | - |  |
| AIMUZH 71 | f | 270.0 | Patagonian | - |  |
| AIMUZH 73 | f | 270.3 | Patagonian | - |  |
| AIMUZH 66 | m | 287.0 | Patagonian | - |  |
| AIMUZH 72 | f | 293.5 | Patagonian | - |  |
| AIMUZH PAL 185 | m | 288.6 | African | - |  |
| AIMUZH PAL 103 | m | 299.7 | African | - |  |
| AIMUZH PAL 227 | m | 308.5 | African | - |  |
| AIMUZH PAL 229 | m | 314.6 | African | - |  |
| AIMUZH PAL 26 | m | 321.0 | African | - |  |
| AIMUZH PAL 224 | m | 325.8 | African | - |  |
| AIMUZH PAL 57 | m | 327.9 | African | - |  |
| AIMUZH PAL 98 | m | 332.5 | African | - |  |
| AIMUZH PAL 153 | m | 333.3 | African | - |  |
| AIMUZH PAL 218 | m | 350.3 | African | - |  |
| AIMUZH PAL 43 | m | 355.5 | African | - |  |
| GAIN9905 | m | 53.1 | neonate | - | 2 |
| AIMUZH6807 | m | 32.2 | - | - |  |
| AIMUZH AS445 | m | 37.1 | - | - |  |
| AIMUZH6830 | f | 37.9 | - | - |  |
| AIMUZH7529 | m | 40.0 | - | - |  |
| AIMUZH13308 | m | 43.4 | - | - |  |
| AIMUZH AS443 | - | 43.5 | - | - |  |
| AIMUZH13302 | f | 44.3 | - | - |  |
| AIMUZH13306 | f | 45.6 | - | - |  |
| AIMUZH13303 | f | 47.4 | - | - |  |
| AIMUZH6866 | m | 47.0 | - | - |  |
| AIMUZH13310 | f | 52.8 | - | - |  |
| AIMUZH AS1666 | m | 52.8 | - | - |  |
| AIMUZH13304 | f | 54.4 | - | - |  |
| AIMUZH5559 | f | 55.1 | - | - |  |
| AIMUZH13301 | m | 60.0 | - | - |  |
| AIMUZH8346 | - | 49.0 | - | - |  |
| AIMUZH9404 | f | 43.6 | - | - |  |
| AIMUZH9361 | f | 51.4 | - | - |  |
| AIMUZH11451 | f | 51.8 | - | - |  |
| AIMUZH7659 | f | 82.0 | - | wild |  |
| AIMUZH AS1813 | m | 86.0 | - | wild |  |
| AIMUZH8606 | m | 88.0 | - | - |  |
| AIMUZH6670 | m | 89.0 | - | captive |  |
| AIMUZH AS1571 | f | 94.0 | - | captive |  |
| AIMUZH PAL4 | f | 97.0 | - | - |  |
| AIMUZH AS1662 | f | 98.0 | - | captive |  |
| AIMUZH10768 | f | 100.0 | - | - |  |
| AIMUZH10742 | m | 103.0 | - | - |  |
| AIMUZH AS1806 | f | 104.0 | - | - |  |
| AIMUZH AS1760 | m | 109.0 | - | captive |  |
| AIMUZH AS1742 | m | 99.0 | - | - |  |
| AIMUZH6695 | m | 108.0 | - | wild |  |
| AIMUZH AS1787 | m | 109.0 | - | captive |  |
| AIMUZH6613 | - | 109.0 | - | wild |  |
| AIMUZH6614 | - | 112.0 | - | wild |  |
| AIMUZH AS1786 | m | 113.0 | - | wild |  |
| AIMUZH6615 | - | 114.0 | - | wild |  |
| AIMUZH7480 | m | 117.0 | - | wild |  |
| AIMUZH PAL221 | m | 117.0 | - | captive |  |
| AIMUZH7421 | f | 121.0 | - | wild |  |
| AIMUZH AS310 | m | 121.0 | - | captive |  |
| AIMUZH AS1755 | f | 125.0 | - | - |  |
| AIMUZH AS1814 | f | 126.0 | - | wild-bo |  |
| AIMUZH7056 | f | 128.0 | - | wild |  |
| AIMUZH7009 | f | 135.0 | - | wild |  |
| AIMUZH AS1808 | m | 143.0 | - | captive |  |
| AIMUZH6972 | m | 147.0 | - | wild |  |
| AIMUZH PAL194 | m | 159.0 | - | captive |  |
| AIMUZH6616 | - | 167.0 | - | wild |  |
| AIMUZH PAL110 | m | 173.0 | - | captive |  |
| AIMUZH AS1687 | f | 178.0 | - | captive |  |
| AIMUZH PAL106 | m | 178.0 | - | captive |  |
| AIMUZH11037 | f | 181.0 | - | - |  |
| AIMUZH AS1785 | m | 197.0 | - | captive |  |
| AIMUZH6938 | m | 198.0 | - | wild |  |
| AIMUZH AS1789 | f | 206.0 | - | captive |  |
| AIMUZH AS1680 | f | 209.0 | - | captive |  |
| AIMUZH PAL96 | f | 197.0 | - | captive |  |
| AIMUZH7078 | f | 198.0 | - | wild |  |
| AIMUZH PAL175 | m | 200.0 | - | captive |  |
| AIMUZH6876 | m | 201.0 | - | wild |  |
| AIMUZH AS1695 | m | 201.0 | - | captive |  |
| AIMUZH AS1586 | f | 212.0 | - | wild |  |
| MRAC153 | f | 190.9 | - | - |  |
| MRAC179 | m | 205.1 | - | - |  |
| MRAC3466 | - | 228.1 | - | - |  |
| MRAC15233 | - | 195.1 | - | - |  |
| MRAC18188 | - | 214.2 | - | - |  |
| USNM398214 | m | 72.3 | - | - | 3 |
| AIMUZH11455 | f | 37.0 | - | - |  |
| AIMUZH10144 | - | 44.0 | - | - |  |
| AIMUZH9290 | - | 45.0 | - | - |  |
| AIMUZH10218 | f | 48.0 | - | - |  |
| AIMUZH11440 | f | 49.0 | - | - |  |
| AIMUZH6674 | f | 58.0 | - | - |  |
| AIMUZH9964 | - | 69.0 | - | - |  |
| AIMUZH7660 | m | 77.0 | - | wild |  |
| AIMUZH7611 | m | 79.0 | - | wild |  |
| AIMUZH9788 | - | 81.0 | - | wild |  |
| AIMUZH8836 | f | 89.0 | - | - |  |
| AIMUZH9965 | - | 91.0 | - | wild |  |
| AIMUZH7129 | f | 94.0 | - | wild |  |
| AIMUZH6612 | - | 100.0 | - | wild |  |
| AIMUZH6611 | - | 101.0 | - | wild |  |
| AIMUZH6939 | - | 103.0 | - | wild |  |
| AIMUZH6845 | m | 106.0 | - | wild |  |
| AIMUZH7123 | m | 107.0 | - | - |  |
| AIMUZH6994 | f | 108.0 | - | wild |  |
| AIMUZH7057 | f | 111.0 | - | wild |  |
| AIMUZH PAL5 | f | 112.0 | - | wild |  |
| AIMUZH6610 | - | 118.0 | - | wild |  |
| AIMUZH6609 | - | 119.0 | - | wild |  |
| AIMUZH AS1696 | f | 121.0 | - | - |  |
| AIMUZH6782 | m | 130.0 | - | wild |  |
| AIMUZH6846 | f | 136.0 | - | wild |  |
| AIMUZH7658 | m | 141.0 | - | wild |  |
| AIMUZH PAL3 | - | 143.0 | - | wild |  |
| AIMUZH7036 | f | 159.0 | - | wild |  |
| AIMUZH6787 | f | 185.0 | - | wild |  |
| AIMUZH6642 | - | 185.0 | - | wild |  |
| AIMUZH7128 | m | 189.0 | - | wild |  |
| AIMUZH PAL2 | f | 189.0 | - | wild |  |
| AIMUZH PAL14 | f | 191.0 | - | wild |  |
| AIMUZH PAL10 | f | 201.0 | - | wild |  |
| AIMUZH PAL6 | f | 202.0 | - | wild |  |
| AIMUZH6788 | f | 208.0 | - | wild |  |
| AIMUZH PAL11 | m | 215.0 | - | wild |  |
| AIMUZH6900 | f | 217.0 | - | wild |  |
| AIMUZH6896 | f | 221.0 | - | wild |  |
| AIMUZH7035 | m | 226.0 | - | wild |  |
| AIMUZH PAL12 | m | 226.0 | - | wild |  |
| AIMUZH PAL8 | m | 238.0 | - | wild |  |
| AIMUZH7011 | m | 241.0 | - | wild |  |
| AIMUZH PAL13 | m | 241.0 | - | wild |  |
| AIMUZH7487 | m | 241.0 | - | wild |  |
| AIMUZH6680 | m | 253.0 | - | wild |  |
| AIMUZH6884 | m | 261.0 | - | wild |  |
| AIMUZH8684 | f | 42.7 | - | - | 4 |
| PRI9732 | m | 40.1 | - | - |  |
| AIMUZH AS1642 | m | 30.9 | - | - |  |
| AIMUZH AS1647 | f | 31.0 | - | - |  |
| AIMUZH AS2142 | m | 31.0 | - | - |  |
| AIMUZH8684 | f | 42.6 | - | - |  |
| AIMUZH AS1603 | f | 43.3 | - | - |  |
| AIMUZH AS1592 | m | 46.9 | - | - |  |
| AIMUZH AS11427 | m | 38.3 | - | - |  |
| APE381 | f | 30.8 | - | - |  |
| AIMUZH AS1591 | f | 62.0 | - | wild |  |
| AIMUZH AS1764 | m | 71.0 | - | wild |  |
| AIMUZH7368 | - | 73.0 | - | - |  |
| AIMUZH AS1590 | m | 74.0 | - | wild |  |
| AIMUZH AS1881 | - | 96.0 | - | - |  |
| AIMUZH AS1607 | - | 98.0 | - | wild |  |
| AIMUZH AS1540 | f | 103.0 | - | wild |  |
| AIMUZH10141 | m | 114.0 | - | captive |  |
| AIMUZH AS1471 | f | 116.0 | - | wild |  |
| AIMUZH AS1767 | f | 120.0 | - | wild |  |
| AIMUZH10129 | f | 123.0 | - | captive |  |
| AIMUZH14741 | - | 130.0 | - | - |  |
| AIMUZH AS1533 | - | 131.0 | - | wild |  |
| AIMUZH AS1553 | m | 134.0 | - | wild |  |
| AIMUZH7278 | f | 134.0 | - | wild |  |
| AIMUZH1574 | f | 148.0 | - | wild |  |
| AIMUZH873 | f | 152.0 | - | - |  |
| AIMUZH AS1472 | m | 153.0 | - | wild |  |
| AIMUZH8685 | f | 164.0 | - | wild-born |  |
| AIMUZH AS1528 | f | 165.0 | - | wild |  |
| AIMUZH1739 | f | 169.0 | - | wild |  |
| AIMUZH AS1531 | m | 171.0 | - | wild |  |
| AIMUZH AS1677 | f | 171.0 | - | captive |  |
| AIMUZH1667 | f | 174.0 | - | - |  |
| AIMUZH AS1529 | m | 180.0 | - | wild |  |
| AIMUZH AS1561 | m | 184.0 | - | wild |  |
| AIMUZH PAL101 | m | 196.0 | - | wild |  |
| AIMUZH AS1077 | m | 200.0 | - | wild |  |
| PRI10489 | m | 34.0 | - | captive | 5 |
| PRI2357 | m | 36.7 | - | captive |  |
| PRI2376 | m | 59.5 | - | captive |  |
| PRI2498 | m | 70.6 | - | captive |  |
| PRI3076 | f | 66.3 | - | captive |  |
| PRI3594 | f | 48.9 | - | captive |  |
| PRI5152 | f | 75.7 | - | captive |  |
| PRI5712 | m | 80.6 | - | captive |  |
| PRI7793 | f | 122.9 | - | captive |  |
| PRI7866 | f | 97.2 | - | captive |  |
| PRI7890 | f | 124.1 | - | captive |  |
| PRI5714 | m | 70.1 | - | captive |  |
| PRI5824 | - | 47.1 | - | captive |  |
| PRI5860 | f | 116.1 | - | captive |  |
| PRI5871 | f | 117.1 | - | captive |  |
| PRI5876 | f | 96.0 | - | captive |  |
| PRI5893 | m | 62.2 | - | captive |  |
| PRI5979 | f | 66.6 | - | captive |  |
| PRI6156 | m | 72.9 | - | captive |  |
| PRI6207 | f | 92.4 | - | captive |  |
| PRI6210 | f | 67.1 | - | captive |  |

Table S2. Coefficients of least square fitting between mean cross-sectional area of femoral diaphysis and PC scores

|  |  | Humans | Chimpanzees | Gorillas | Orangutans | Japanese macaques |
| --- | --- | --- | --- | --- | --- | --- |
| PC1 | *R* | 0.776 | 0.832 | 0.661 | 0.667 | 0.872 |
|  | *p* | 0.000 | 0.000 | 0.000 | 0.000 | 0.000 |
| PC2 | *R* | -0.656 | 0.043 | 0.540 | 0.327 | -0.515 |
|  | *p* | 0.000 | 0.724 | 0.000 | 0.045 | 0.017 |
| PC3 | *R* | 0.670 | 0.251 | 0.666 | 0.636 | 0.398 |
|  | *p* | 0.000 | 0.036 | 0.000 | 0.000 | 0.074 |
